# Body Mass Index and Facial Cues in Sasang Typology for Young and Elderly Persons

**DOI:** 10.1155/2011/749209

**Published:** 2011-02-22

**Authors:** Duong Duc Pham, Jun-Hyeong Do, Boncho Ku, Hae Jung Lee, Honggie Kim, Jong Yeol Kim

**Affiliations:** ^1^Department of Medical Research, Korea Institute of Oriental Medicine (KIOM), 461-24 Jeonmin-dong, Yuseong-gu, Daejeon 305-811, Republic of Korea; ^2^Department of Korean Traditional Medicine and Biotechnology, University of Science and Technology, 176 Gajung-dong, 217 Gajung-ro, Yuseong-gu, Daejeon 305-350, Republic of Korea; ^3^Department of Internal Medicine, National Hospital of Traditional Medicine, 29 Nguyen Binh Khiem, Hanoi 112611, Vietnam; ^4^Department of Information and Statistic, Chungnam National University, 79 Daehang-ro, Yuseong-gu, Daejeon 305-764, Republic of Korea

## Abstract

Facial characteristics may provide reliable information giving an insight into the inner nature of an individual. This study examines the differences in widely used facial metrics, including cheek-to-jaw width ratio (CJWR), width-to-height ratio (WHR), perimeter-to-area ratio (PAR), and facial masculinity indexes across Sasang constitutional types, to investigate the association between these facial cues and body mass index (BMI) and develop a predictive model for Sasang typing. 2D images of 911 participants were analyzed. The results indicated that TaeEum (TE) type generally has a squarer face, with the male TE type having a squarer and wider face than that of both SoYang (SY) and SoEum (SE) types. Male TE type has longer eyes than that of the SE type, and the lower face of the female TE type is longer than that of the SY type. PAR, WHR, CJWR, and eye size had associations with BMI, and the magnitude of correlation of CJWR in Korean men were twofold higher than that of the Caucasian and African men. BMI and facial metrics including PAR, WHR, CJWR, and eye size were good predictors for TE type, and the most parsimonious model for TE typing included BMI and CJWR with high predictive performances.

## 1. Introduction

The human face containing cues of gender, ethnicity, attractiveness, emotions, personality traits, and so on has been the subject of speculation for centuries [1]. In the West, physiognomy, the theory used to assess a person's nature and personality via external appearance and face, was first mentioned by ancient Greek philosophers and was later described in detail by Johann Kaspar Lavater (1741–1801) [2]. Meanwhile, in the East, particular facial characteristics were believed to refer to assessable information of an individual's personality and destiny (according to “face reading” theory), as well as health status (as stated by traditional Chinese medicine) [3, 4]. These theories seem to be fanciful, and they are criticized from a scientific viewpoint. 

However, scientific evidences found recently indicate that the human face can provide reliable information to understand the inner nature of a person. One approach focuses on facial symmetry [5–8], averageness [6, 7], sexual dimorphism [8, 9], and skin color/texture [10, 11] as cues to attractiveness, the perceived information affecting mate choice preferences. There has been an effort to investigate the link between facial attractiveness and health, but the outcomes appeared to be ambiguous. Numerous studies have revealed that facial attractiveness is correlated with longevity [12], reproductive health [13, 14] as well as some physical illness symptoms (e.g., runny nose, nausea, backache, etc.) [15] and heterozygous human leukocyte antigen (HLA) genes [16], whereas other studies found no relationship between facial attractiveness and the history of acquired infectious bouts and the use of antibiotics [17]. 

Recent approach has relied on more proximate and quantifiable facial cues to judge health. It has been demonstrated that facial adiposity, the perception of weight in the face, is associated with perceived health and attractiveness in a curvilinear relationship, whereas there is a linear correlation between facial adiposity and blood pressure [18]. Coetzee et al. found that three facial metrics (width-to-height ratio (WHR), cheek-to-jaw-width ratio (CJWR), and perimeter to area ratio (PAR)) are associated with body mass index (BMI) in males and WHR and CJRW are correlated with BMI in female [19]. Studies with a psychological perspective reported that WHR might be considered as an “honest signal” of propensity for aggressive behavior and people with a larger WHR act more aggressively than those with a smaller WHR [20, 21]. A recent study reported that facial masculinity that relied on the measurement of sexually dimorphic facial traits is associated with the variation of testosterone concentration after a competitive task [22]. These evidences suggest that subtle differences in the human face can provide valuable information giving an insight into the diversity of our nature. In other words, the propensity toward particular personality traits, social judgments, and health status might be predicted by analyzing particular facial cues. 

People differ from each other not only in facial appearance but also in inherited genetic information that determine an individual's personality traits, predisposition to certain kinds of diseases, and reaction to certain drugs. It is plausible to assume that there may be particular human types or human constitutions in which the aforementioned distinctions are encoded and inherited. There are several well-known theories of constitutional medicine including the “Four Humors” theory of Hippocrates and Galen, the three categories of body shape by the modern scientists Kretschmer and Sheldon [23], the constitution-based theory of Ayurveda [24], and the Sasang typology [25]. 

Sasang constitutional medicine (SCM), founded by Jema Lee (1894), is a unique form of traditional Korean medicine that emphasizes the distinctive tendencies of physiological and pathological states among individuals. SCM classifies human beings into four constitutional types, TaeYang (TY), SoYang (SY), TaeEum (TE), and SoEum (SE) types, differing in terms of (i) physical features, (ii) psychological characteristics, (iii) susceptibility to certain health problem patterns, and (iv) response to certain herbs and medicine [25, 26]. According to the SCM theory, determination of an individual's constitution based on body shape analysis, facial cue, voice analysis, along with a questionnaire is the primary task in the treatment of an individual with a constitutional approach [27]. Although facial characteristics were not mentioned in Lee's original textbook, his successors have developed investigative methods on facial cues for constitutional typing. TY types are characterized with a big and round face, a wide forehead, big ears, and a high bridge, whereas TE types are described as persons with a wide jaw, wide distance between eyebrows, big eyes, thick ears, and thick lips. SY types have a small, round, and bulging face, full-of-spirit eyes, high and sharp nose, and thin and small lips, whereas SE types have an ellipsoid or round face, a little predominant forehead, low upper eyelids and corners, big earlobes, a small nose, and a big mouth [28]. Several attempts have been made to investigate the typical facial features of each SCM type using quantitative measurements on 2D and 3D images [28–31]. These studies have indicated that the SCM types differ in several facial features such as length of jaw, length of face, and shape of eyes. However, the widely used facial metrics for symmetry, sexual dimorphism, and facial masculinity including WHR, CJWR, PAR, and facial masculinity indexes have not been employed in Sasang typing so far. Although it has been demonstrated that WHR, CJWR, and PAR are correlated to BMI in Caucasian and African young people [19], such associations have yet to be studied in Asian ethnic groups. As BMI has been reported as a good predictor for Sasang typing [32–34], especially TE type, a model that consists of BMI and facial metrics may be a promising tool for the determination of Sasang types. Because facial features are influenced by age and gender, these factors need to be taken into account in a facial metric study [35]. 

The aim of this study was to investigate the distinctive facial characteristics of Sasang types based on widely used facial metrics such as facial masculinity indexes, WHR, CJWR, and PAR, and to evaluate the association of these variables with BMI in young and elderly Korean people. Secondly, we sought to develop a model for predicting the probability of Sasang types using BMI and these facial cues.

## 2. Methods

### 2.1. Participants

Participants numbering 1566 in their twenties and sixties were recruited from March to September 2009 from the Korea Institute of Oriental Medicine (KIOM) via advertisement to obtain information for the Sasang Constitutional Bank project, a national project to investigate the distinctive characteristics of Sasang types. Participants were recruited from 9 colleges and 8 welfare centers in Daejeon city, one of the five biggest cities in the Republic of Korea. The IRB of KIOM (no. I0903-01-02) approved this study and the participants gave their written consents.

Two experienced SCM specialists having a minimum of 5 years working experience in the field of SCM independently determined the constitution of these subjects by clinical examinations based on SCM theory. SCM specialists were asked to strictly follow a standardized operation procedure (SOP) and case report form (CRF). Clinical determination of each SCM constitutional type was classified in three confidence levels, high, medium, and low. The subjects who had a certain constitutional determination with high confidence level and confirmed by both specialists were selected [36]. 

After Sasang typing, 553 participants were excluded because their Sasang types were not determinable, whereas 1013 participants entered the steps of data collection including facial images and voice and pulse analysis. The prevalence of the TY type is extremely low as described in the literature [25] and there were only six TY persons in the present study; we therefore focused only on three Sasang types (TE, SE, and SY types). 96 persons were then excluded because of erroneous photo and data encoding operations. As such, the final data of facial analysis and BMI of 911 participants including TE (in their twenties *n* = 189, in their sixties *n* = 177), SE (in their twenties *n* = 111, in their sixties *n* = 111), and SY (in their twenties *n* = 159, in their sixties *n* = 164) types were analyzed.

### 2.2. Photographs

Participants were photographed with a neutral expression, under standard conditions (using Nikon D700 with 85 mm lens, distance between the camera and the participant was 1.6 m, bilateral illumination, hair was pulled backward with hair band to reveal the hair line). Participants were required to look directly into the camera and their heads were postured up straight so that the central point of the two pupils and the two points defined by the connections between facial contour and upper auricular perimeters were lined on the same horizontal line. Images were captured at a resolution of 3184 × 2120 pixels in JPEG format using 24-bit RGB encoding.

### 2.3. Measurements

**Figure 1:**
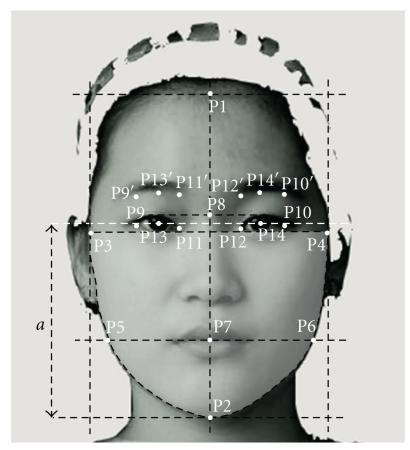
Points used in the calculation of facial metric measurements.

Each facial image was manually assigned with 20 points (Figure 1). The measurements such as PAR, WHR, and CJWR was computed following the procedure of Coetzee et al. [19], and the measurements of facial masculinity indexes were taken following the procedure of Penton-Voak et al. [8]. 

Perimeter-to-area ratio was defined as the ratio of the length of a polygon running through P3, P5, P2, P6, P4, and P3 to the shaded area within the perimeter. With the aid of 2D images, it was determined that the lower the PAR the rounder the circularity. WHR was defined as the ratio of cheekbone width, that is, the distance between P3 and P4, divided by the distance between P7 and P8. CJWR was calculated as the ratio of cheekbone width to the jaw width, the distance between P5 and P6. A larger WHR and a smaller CJWR indicate a wider and a squarer face, respectively. Components of facial masculinity indexes were five facial metrics: (i) CJWR, (ii) eye size (distance between P9 and P10 minus the distance between P11 and P12 divided by two), (iii) lower face to face height ratio (LF/FH) (the distance *a* between the horizontal line through P2 and the horizontal line through two pupils divided by the distance between P1 and P2), (iv) face width to lower face height ratio (FW/LFH) (the distance between P3 and P4 divided by the distance *a*), and (v) mean of eyebrow height (mean of distance between P9 to P9′, P10 to P10′, P11 to P11′, P12 to P12′, P13 to P13′, and P14 to P14′). A high score of these indexes indicates a high masculinity. 

Point assignments and distance measurements were done based on self-made software using MATLAB language on a Window XP platform. Point assignments were done by a well-trained operator who was blind to the determined constitutions of participants, and the test-retest on 15 randomized photos showed high reliability with low coefficient of variation (0.4~5.53%). 

Body weight and height were measured using a digital scale (GL-150; G Tech International Co., Ltd, Uijeongbu, Republic of Korea) while participants wore a casual suit without shoes and their posture was straight with the mandible plane parallel to the floor. BMI was calculated as weight (kg) divided by the square of height (m).

### 2.4. Data Analysis

As the facial characteristics and BMI are influenced by age and gender [35], data analysis was conducted separately for each group of age and gender. Our primary objective was to use facial cues to distinguish Sasang types and to evaluate the correlation of facial metrics to BMI. Differences of facial metrics and BMI across Sasang types were investigated using the one-way ANOVA test, in which the differences between the groups were analyzed by the Duncan test. Pearson correlation coefficient was calculated to assess the association of facial metrics to BMI. We then compared the magnitude of correlation of PAR, CJWR, and WHR to the BMI of the present study with that of the results obtained in the study by Coetzee et al. [19] conducted on young Caucasians and Africans. 

The secondary objective of the present study was to develop a diagnostic model for predicting the probability of Sasang types in an individual using BMI and facial metrics. Based on the differences in BMI and measured facial metrics across Sasang types, we decided the target Sasang type for developing a diagnostic predictive model. We used logistic regression analysis to assess the relationship of BMI and facial cues to the target Sasang type. Given multiple possible predictors including BMI and seven facial metrics, the number of models that needed to be analyzed was 2^8^. We used the Bayesian model average approach to obtain the most parsimonious model that included minimum number of predictive factors with maximum discriminatory powers. The most parsimonious model is the model with the lowest Bayesian information criterion value (BIC). Based on the obtained model, a predictive equation was created to estimate the probability of the target Sasang type with a given value of predictive factors included in the model. The predictive performance of the parsimonious model was assessed by *c* statistic value that was estimated by the area under the receiver operating characteristic (ROC) curve. The higher value of the *c* statistic implies better predictive performance [37]. All analyses were conducted using R language on a Window XP platform [38].

## 3. Results

### 3.1. Demographic Characteristic and Anthropometric Profile

Data were analyzed separately based on age and gender groups (i) male-twenties (M20s), (ii) male-sixties (M60s), female-twenties (F20s), and female-sixties (F60s) (Tables 1 and 2). These four groups were comparable in age and height. The age of the TE participants was lower than that of the SE and SY participants in the F20s group. There were consistent differences in body weight and BMI among the Sasang types in the four age- and gender-based groups (M20s: *P* = .000; M60s: *P* = .000; F20s: *P* = .000; F60s: *P* = .000). Post hoc tests indicated that the TE type had higher BMI and body weight than that of the SY type, and the value of these measurements of the SE type was lowest significantly. The magnitude of differences in BMI between the TE type and the SE type of male groups was higher than that of the female group.

### 3.2. Facial Cues

There were consistent differences in CJWR among the Sasang types in all four age-gender-based groups (M20s: *P* = .000; M60s: *P* = .000; F20: *P* = .001; F60s: *P* = .000). Post hoc tests revealed that CJWR of the TE type was lower than that of the SY and SE types in M20s, F20s, and F60s groups, whereas in M60s group, the TE type had the lowest CJWR followed by the SY type, and the SE type had the highest CJWR. 

In male groups, the value of WHR in the TE type was higher than that of the SE and SY types (M20s: *P* = .002, M60s: *P* = .000), whereas no difference was found in the female groups. Moreover, the post hoc test showed that the TE type had larger eye size (M20s: 32.60 ± 1.79; M60s: 29.62 ± 1.96) than the SE type (M20s: 31.66 ± 1.80; M60s: 28.70 ± 2.34), and SY shared similarities to both the TE and SE types. 

In female groups, the post hoc test indicated that the value of LF/FH of the TE type (F20s: 0.61 ± 0.02; F60s: 0.63 ± 0.02) was higher than that of the SY type (F20s: 0.60 ± 0.02; F60s: 0.62 ± 0.02).

The value of PAR was found to be lower in the TE type in comparison with the SE type in M20s and F60s groups, and the SY type in F20s group had a higher FW/LF than the TE and SE types. No difference was found for the EH mean.

### 3.3. Correlation of Facial Metrics to BMI

Pearson's correlation test indicated that in the four age-gender based groups, PAR and CJWR had a negative correlation with BMI, whereas eye size correlated positively with BMI (Table 3), in which the magnitude of correlation of CJWR to BMI appeared to be the highest. WHR was correlated positively with BMI in three of the four age-gender based groups (M20s: *r* = 0.3, M60s: *r* = 0.28, and F20s: *r* = 0.28), whereas no correlation was found in the F60s group. LF/FH was correlated positively with BMI in only the F60s groups. 

Coetzee et al. conducted a study on Caucasian and African participants who were in their twenties and found that CJWR had a negative correlation with BMI, WHR was correlated positively with BMI in male and female groups, and PAR had a negative correlation with BMI in male groups only [19]. This finding is in line with our results; however, we have questioned the differences of magnitude of these correlations in the Caucasians and Africans done by Coetzee et al. with those in Asians, especially Koreans, in the present study.

We applied Fisher's z transformation to compare the magnitude of correlation coefficients and to derive their significance [39]. The result in Table 3 shows that in the present study, the magnitude of correlation of PAR, CJWR, WHR, and eye size to BMI in the young group was similar to that in the old groups, except that WHR correlated with BMI in the young female group but not in the old female group. In the comparison between the present study and that of Coetzee et al., the magnitude of correlation of PAR, CJWR, and WHR to BMI in the young Korean people was similar to that in the young Caucasian and African people, except that the magnitude of correlation of CJWR to BMI in the Korean male groups was two times higher than in the male Caucasian and African people (*P* value =  .03).

### 3.4. Diagnostic Predictive Model for TE Type

Results, in Tables 1 and 2, show that there are significant differences in facial metrics between TE type and non-TE types (SE and SY types) of Sasang typology. Therefore, we have developed a predictive model for the determination of the TE type. Because body weight had a strong correlation to BMI (*r* = 1) (data not shown) and BMI is an important predictor for the TE type [32–34], the potential predictive factors were BMI and the aforementioned facial metrics.

 Logistic regression analysis (Table 4) suggests that BMI and CJWR were the most important predictive factors for the TE type. Each one unit increase in BMI was associated with an increase in an odd ratio of 1.63 (95% CI 1.42–1.88) in the M20s group, 1.52 (1.33–1.74) in the M60s group, 1.62 (1.39–1.89) in the F20s group, and 1.79 (1.51–2.11) in the F60s groups. Each SD (0.05) increase in CJWR was associated with a decrease in an odd ratio of 0.55 (95% CI 0.37–0.67) in M20s group, 0.65 (0.49–0.76) in M60s group, 0.41 (0.2–0.56) in F20s group, and 0.48 (0.3–0.62) in F60s groups. The result of the c statistic value indicated that BMI and facial metrics, especially PAR, CJWR, WHR, and eye size, were related to the TE type.

**Figure 2:**
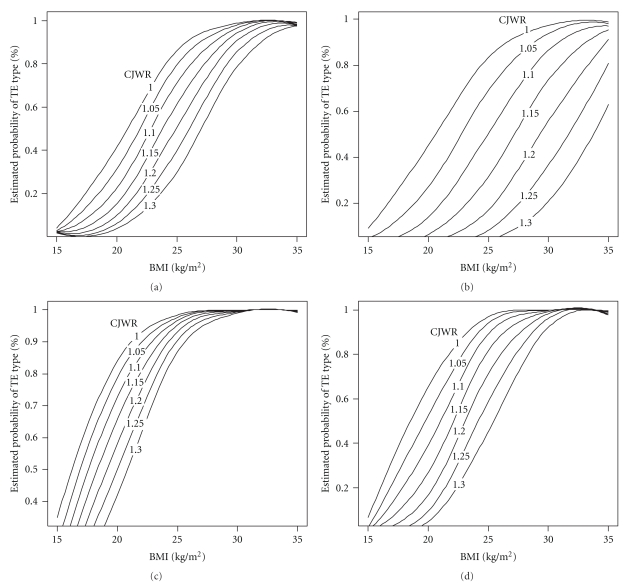
Predicted probability of TE type in twenties men (a), sixties men (b), twenties women (c), and sixties women (d) for a given BMI and CJWR.

Bayesian model average analysis suggested that the model consisting of BMI and CJWR was the most parsimonious model in the four age-gender based groups, in which each one unit increase in BMI was associated with 1.57-fold (95% CI 1.36–1.82), 1.45-fold (1.26–1.67), 1.58-fold (1.35–1.85), and 1.72-fold (1.45–2.04) increase in the odd ratio of the TE type in the M20s, M60s, F20s, and F60s, respectively. The decrease in odd ratio of 0.37-fold (0.09–0.57), 0.56-fold (0.33–0.71), 0.3-fold (0.16–0.51), and 0.46-fold (0.17–0.65) in M20s, M60s, F20s, and F60s, respectively, was associated with each 0.05 increase in CJWR. The estimated probability of the TE type was calculated according to the following equation: probability = 1/(1 + e^*x*^), in which *x* = 0.236 − 0.454BMI + 9.237CJWR for M20s group, *x* = −8.572 − 0.373BMI + 16.254CJWR for M60s group, *x* = 0.556 − 0.4617BMI + 6.9579CJWR for F20s group, and *x* = −1.77824 − 0.54430BMI + 11.99360CJWR for F60s group (Figures 2(a), 2(b), 2(c), and 2(d)). The areas under the ROC curve of these predictive models were 0.82, 0.82, 0.78, and 0.83 for M20s, M60s, F20s, and F60s, respectively. Internal validation was evaluated using the cross-validation approach, and the results showed that the cross-validated correct rate of these predictive models were 76.1 (error 0%), 72.9 (1.4%), 71.2 (0.4%), and 70.9 (0.9%) for M20s, M60s, F20s, and F60s, respectively. In this model, the interaction effect between two variables (BMI and CJWR) was not significant.

## 4. Discussion

Deciphering human faces has been an issue of interest since ancient times. Recent quantitative and standardized studies on facial cues have suggested that individuals differ in certain facial structural characteristics and these features may be reliable signals of psychological and physical patterns [5–22]. The present study indicated that several facial metrics that are commonly used in the study of facial structure could be employed to distinguish the Sasang constitutional types, especially the TE type. Data showed that CJWR was significantly lower in the TE type in every age-gender-based group and WHR was larger in the TE type in male groups. A larger WHR implies a wider face and a smaller CJWR indicates a squarer face as discussed by Coetzee et al. [19]. In addition, the eye size of the male TE type was wider significantly in comparison with the male SE type. In female groups, LF/FH of the TE type was higher than that of the SY type. A higher LF/FH indicates a longer lower face. 

 This result suggested that (i) the TE type has a squarer face generally, (ii) the male TE type has a squarer and wider face than both the SY and SE types, (iii) male TE type has longer eyes than the SE type, and (iv) the lower face of the female TE type was longer than that of the SY type. No previous study uses the facial metrics used in the present study on Sasang typology; however, previous studies reported results somewhat compatible with this study. Several studies have revealed that the TE type has a wider jaw [28, 29], the SE type has a smaller face [28], and the eyes of the SY and SE types are rounder than those of the TE type. However, these studies did not take into account the influences of gender and age factors on the differences of the facial cues across Sasang types. Yoon et al. investigated that the elderly TE men had wider face, whereas the TE women had wider and longer face [31]. The facial features used to discriminate the SY and SE types were inconsistent from study to study. Koh et al. suggested that the shape of the eyes [29] distinguished the SY and SE types, whereas Lee et al. focused on the facial length [28] and Yoon et al. employed the features of the noses and eyebrow [31]. In the present study, the SE and SY types shared similarities in every facial metric measurement. 

Because the TE type is closely associated with high BMI, we questioned whether our measured facial metrics were correlated with the health variable. We found that PAR, WHR, CJWR, and eye size had associations with BMI in every age-gender based group, except WHR in the F60s group, and the magnitude of these associations in the young and old groups were not different. This result was compatible to the finding in the Caucasian and African people [19]; however, the magnitude of correlation of CJWR in the Korean men was twofold higher than that in the Caucasian and African men. Based on this finding, Coetzee suggested that fatter persons have higher buccal fat and masseter muscle volumes; therefore, they have a lower value of PAR and CJWR. It was also hypothesized that heavier persons have bigger frame size, and consequently have higher WHR [19]. In the present study, we also found that eye size, a component of masculinity index, was associated with BMI and we assumed the fatter person to have a bigger orbit. It is plausible to assume that higher volume of buccal fat and masseter muscle and bigger orbit may be the distinctive characteristics of the TE type, and future studies should measure these variables directly. 

The next aim of this study was developing a predictive model to distinguish three constitutions (TE, SE, and SY). However, after conducting ANOVA analysis we could find only the differences among the TE type and non-TE type (SE and SY types), therefore the logistic regression analysis for binary endpoint was employed. Since the population of the TY type is extremely low and constitutional undeterminable persons may belong to one of four SCM types, this analysis did not take into account those individuals. 

We used the Bayesian model average approach, which is demonstrated to provide better accuracy and less bias than conventional approaches, to develop a diagnostic predictive model to discriminate the TE type from the other Sasang types. Because facial metrics correlated to BMI, we included BMI into the list of potential predictive factors for logistic regression and Bayesian model average analysis to investigate whether BMI only could be used as the best predictor to the TE type and to determine the parsimonious model for TE typing. We found that BMI and measured facial metrics, especially PAR, WHR, CJWR, and eye size, influenced the determination of the TE type, and the most parsimonious model for TE typing included BMI and CJWR.

Numerous studies have revealed that high BMI is one of the distinctive characteristics of the TE type [32–34]; however, there has been no predictive model developed to discriminate the TE type. Previous studies on facial images have demonstrated that the width of jaw is an important variable and it could be regarded as a crucial feature to distinguish the TE type [28, 29]. A person with wider jaw has lower CJWR, and the width of jaw is not only defined by buccal fat and masseter muscle volumes, but also by the mandible size. In the present study, we found that among measured facial metrics, CJWR was significantly different across the Sasang types in every age-gender-based group and had the highest magnitude of association with the probability of the TE type. Bayesian model average analysis indicated that the BMI only was insufficient to describe the variation of TE type. The model using BMI and CJWR had a relatively high predictive performance with the area under the ROC curve 0.82, 0.82, 0.78, and 0.83 for M20s, M60s, F20s, and F60s, respectively, and the error was relatively low (0% for M20s, 1.4% for M60s, 0.4% for F20s, and 0.9% for F60s). This is the first predictive model to discriminate the TE type so far. 

The present findings must be interpreted in the context of their strengths and potential limitations. The strength of this study is that age and gender factors were taken into account and analyzed separately, which provided explicit pictures of distinctive facial features across the Sasang types. Because the sample size of the spresent study is large enough, participants were divided into four subgroups, and this approach usually provides more accurate result than an analysis using covariates. The result was not different when we analyzed using age and gender as covariates. Facial images were photographed in a standardized condition, which minimized the bias of data collection. The Bayesian model average analysis that was employed, overcame the ignorance of the unselected variables and the uncertainty of the conventional stepwise strategy, and therefore, it provided a more accurate predictive model [40]. However, as the Sasang typing relied on clinical determination by Sasang specialists, there may be a diagnostic bias that should be minimized by a set of diagnostic tools such as a questionnaire, herbal responses, and clinical examination. As this study focused on young (twenties group) and old (sixties group) individuals, the result may not be generalized for middle-aged people. 

In conclusion, these data indicate that the Sasang types have distinctive facial features such as PAR, WHR, CJWR and eye size, which correlates with BMI. They also show that BMI and CJWR can be used to discriminate the TE type from the SY and SE types in Sasang typing.

## Figures and Tables

**Table 1:** Demographic characteristic, anthropometric profile, and facial metrics in twenties groups.

	M20s				F20s			
	TE (*n* = 96)	SE (*n* = 57)	SY (*n* = 77)	*P* value	TE (*n* = 93)	SE (*n* = 54)	SY (*n* = 82)	*P* value
Age (y)	24.0 (2.4)	23.4 (2.4)	23.2 (2.5)	.074	21.4 (1.7)^b^	23.0 (2.4)^a^	22.4 (2.0)^a^	.000
Height (m)	175.4 (5.2)	173.9 (5.3)	174.6 (5.2)	.142.	161.6 (4.9)	160.2 (5.1)	162.2 (5.2)	.096
Weight (kg)	76.1 (10.4)^a^	62.8 (6.4)^c^	67.6 (6.3)^b^	.000	58.6 (6.9)^a^	50.4 (6.6)^c^	52.6 (5.9)^b^	.000
BMI (kg/m^2^)	25.1 (3.4)^a^	20.7 (2.1)^c^	22.3 (2.0)^b^	.000	19.35 (2.28)^a^	16.63 (2.18)^c^	17.38 (1.94)^b^	.000
PAR	0.045 (0.002)^b^	0.046 (0.002)^a^	0.046(0.0018)^a^	.002	0.047 (0.002)	0.048 (0.002)	0.048 (0.002)	.115
WHR	2.16 (0.14)^a^	2.08 (0.13)^b^	2.11 (0.14)^b^	.002	2.18 (0.13)	2.14 (0.13)	2.15 (0.13)	.169
CJWR	1.13 (0.05)^b^	1.17 (0.05)^a^	1.16 (0.05)^a^	.000	1.16 (0.04)^b^	1.19 (0.05)^a^	1.18 (0.05)^a^	.001
Eye size (mm)	32.60 (1.79)^a^	31.66 (1.80)^b^	32.19 (1.72)^ab^	.007	31.72 (1.73)	31.16 (1.77)	31.52 (1.60)	.161
LF/FH	0.63 (0.03)	0.62 (0.03)	0.62 (0.02)	.185	0.61 (0.02)^a^	0.60 (0.02)^ab^	0.60 (0.02)^b^	.037
FW/LF	1.21 (0.06)	1.19 (0.07)	1.19 (0.06)	.162	1.23 (0.05)^b^	1.23 (0.05)^b^	1.25 (0.05)^a^	.022
EH mean (mm)	16.58 (2.11)	16.22 (1.86)	16.63 (1.68)	.435	17.92 (1.85)	17.82 (1.97)	18.04 (2.06)	.803

Value is presented as mean (SD). *P* value: ANOVA test result.

^a,b,c^Significant difference between groups in which value descends respectively by Duncan test.

M20s: male-twenties group; F20s: female-twenties group, TE: TaeEum type, SE: SoEum type, and SY: SoYang type.

**Table 2:** Demographic characteristic, anthropometric profile, and facial metrics in sixties groups.

	M60s				F60s			
	TE (*n* = 87)	SE (*n* = 51)	SY (*n* = 80)	*P* value	TE (*n* = 90)	SE (*n* = 60)	SY (*n* = 84)	*P* value
Age (y)	65.0 (3.4)	65.5 (3.6)	65.8 (3.3)	.339	64.9 (3.1)	63.8 (2.9)	64.1 (3.0)	.086
Height (m)	166.8 (5.9)	166.4 (5.5)	165.5 (5.2)	.229	154.6 (4.8)	153.3 (4.7)	153.4 (4.4)	.133
Weight (kg)	73.2 (8.3)^a^	60.5 (8.0)^c^	66.2 (7.9)^b^	.000	63.7 (6.0)^a^	53.1 (6.1)^c^	57.9 (6.3)^b^	.000
BMI (kg/m^2^)	24.18 (2.74)^a^	19.99 (2.66)^c^	21.88 (2.60)^b^	.000	21.03 (1.97)^a^	17.54 (2.03)^c^	19.13 (2.08)^b^	.000
PAR	0.045 (0.002)	0.045 (0.002)	0.045 (0.002)	.789	0.047 (0.002)^b^	0.048 (0.003)^a^	0.047 (0.003)^ab^	.018
WHR	2.08 (0.14)^a^	1.99 (0.13)^b^	2.02 (0.11)^b^	.000	2.13 (0.15)	2.12 (0.13)	2.12 (0.16)	.951
CJWR	1.06 (0.04)^c^	1.11 (0.05)^a^	1.09 (0.04)^b^	.000	1.07 (0.04)^b^	1.10 (0.04)^a^	1.09 (0.04)^a^	.000
Eye size (mm)	29.62 (1.96)^a^	28.70 (2.34)^b^	29.06 (2.03)^ab^	.032	28.99 (1.86)	28.22 (2.40)	28.90 (1.94)	.059
LF/FH	0.62 (0.02)	0.61 (0.02)	0.62 (0.02)	.718	0.63 (0.02)^a^	0.62 (0.02)^b^	0.62 (0.02)^b^	.003
FW/LF	1.21 (0.06)	1.19 (0.06)	1.20 (0.05)	.085	1.21 (0.06)	1.22 (0.05)	1.21 (0.06)	.802
EH mean (mm)	18.29 (2.57)	17.72 (3.12)	18.28 (2.98)	.464	20.28 (3.18)	19.90 (2.87)	19.47 (2.76)	.200

Value is presented as mean (SD). *P* value: ANOVA test result.

^a,b,c^Significant difference between groups in which value descends respectively by Duncan test.

M20s: male-twenties group; F20s: female-twenties group, TE: TaeEum type, SE: SoEum type, and SY: SoYang type.

**Table 3:** Correlation between BMI and facial cues.

	20s^r^		60s^r^		Coetzee et al. [19]^r^ (age: 20s)		*P* value (Pham 20s versus Coetzee)		*P* value (Pham 20s versus Pham 60s)	
	Male (*n* = 230)	Female (*n* = 229)	Male (*n* = 219)	Female (*n* = 234)	Male (*n* = 187)	Female (*n* = 194)	Male	Female	Male	Female
PAR	−0.30***	− 0.23***	− 0.18**	− 0.28***	− 0.22**	− 0.12	0.39	0.25	0.18	0.57
WHR	0.30***	0.28***	0.28***	0.06	0.17*	0.36***	0.16	0.36	0.82	2.43
CJWR	− 0.40***	− 0.29***	− 0.40***	− 0.27***	− 0.20**	− 0.29***	0.03	1	1	0.82
Eye size	0.29***	0.26***	0.27***	0.21**					0.82	0.57
LF/FH	0.12	0.10	0.07	0.29***						
FW/LF	0.12	0.05	0.16*	0.052						
EH mean	0.05	0.16*	0.11	0.12						

^r^Pearson's correlation coefficient; *P < .05, **P < .01, ***P < .001. 20s: twenties group; 60s: sixties group.

*P* value calculated by Fisher's z transformation approach, in which *P* < .05 indicates the significant difference of magnitude of two correlation coefficients.

**Table 4:** BMI and facial metrics in association with TE types: logistic regression analysis.

	Comparison unit	M20s		M60s		F20s		F60s	
		OR (CI 95%)	*c* statistic	OR (CI 95%)	*c* statistic	OR (CI 95%)	*c* statistic	OR (CI 95%)	*c* statistic
BMI	1	1.63 (1.42–1.88)	0.81	1.52 (1.33–1.74)	0.78	1.62 (1.39–1.89)	0.77	1.79 (1.51–2.11)	0.82
PAR	0.002^sd^	0.61 (0.46–0.81)	0.63	0.91 (0.70–1.20)	0.53	0.75 (0.57–0.99)	0.58	0.72 (0.54–0.95)	0.58
WHR	0.14^sd^	1.60 (1.21–2.12)	0.64	1.74 (1.28– 2.35)	0.65	1.28 (0.98–1.67)	0.57	1.04 (0.8–1.35)	0.54
CJWR	0.05^sd^	0.45 (0.33–0.63)	0.69	0.35 (0.24–0.51)	0.74	0.59 (0.44–0.80)	0.64	0.52 (0.38–0.70)	0.67
Eye size	1	1.22 (1.05–1.42)	0.60	1.18 (1.03– 1.35)	0.61	1.13 (0.96–1.32)	0.56	1.09 (0.96–1.25)	0.55
LF-FH	0.02^sd^	1.27 (0.98–1.66)	0.56	1.09 (0.83–1.43)	0.53	1.42 (1.08–1.86)	0.60	1.57 (1.19–2.07)	0.62
FW-LF	0.06^sd^	1.29 (0.99–1.69)	0.58	1.30 (0.98–1.71)	0.57	0.82 (0.63–1.07)	0.45	0.95 (0.73–1.24)	0.48
EH mean	1	1.03 (0.9–1.19)	0.51	1.03 (0.94–1.13)	0.52	0.99 (0.86–1.13)	0.51	1.08 (0.98–1.18)	0.57

M20s: male-twenties group; F20s: female-twenties group; M60s: male-sixties group; F60s: female-sixties group.

^sd^The comparison unit was adjusted to be close to the standard deviation of each potential predictive factors.

## References

[1] Zebrowitz LA (2006). Finally, faces find favor. *Social Cognition*.

[2] Erle S Face to Face with Johann Caspar Lavater.

[3] Yap J (2008). *Face Reading: The Chinese Art of Physiognomy*.

[4] Bridges L (2003). *Face Reading in Chinese Medicine*.

[5] Grammer K, Thornhill R (1994). Human (Homo sapiens) facial attractiveness and sexual selection: the role of symmetry and averageness. *Journal of Comparative Psychology*.

[6] Rhodes G, Zebrowitz LA, Clark A, Kalick SM, Hightower A, McKay R (2001). Do facial averageness and symmetry signal health?. *Evolution and Human Behavior*.

[7] Rhodes G, Yoshikawa S, Palermo R (2007). Perceived health contributes to the attractiveness of facial symmetry, averageness, and sexual dimorphism. *Perception*.

[8] Penton-Voak IS, Jones BC, Little AC (2001). Symmetry, sexual dimorphism in facial proportions and male facial attractiveness. *Proceedings of the Royal Society B*.

[9] Perrett DI, Lee KJ, Penton-Voak I (1998). Effects of sexual dimorphism on facial attractiveness. *Nature*.

[10] Fink B, Grammer K, Thornhill R (2001). Human (Homo sapiens) facial attractiveness in relation to skin texture and color. *Journal of Comparative Psychology*.

[11] Jones BC, Little AC, Feinberg DR, Penton-Voak IS, Tiddeman BP, Perrett DI (2004). The relationship between shape symmetry and perceived skin condition in male facial attractiveness. *Evolution and Human Behavior*.

[12] Henderson JJA, Anglin JM (2003). Facial attractiveness predicts longevity. *Evolution and Human Behavior*.

[13] Soler C, Núñez M, Gutiérrez R (2003). Facial attractiveness in men provides clues to semen quality. *Evolution and Human Behavior*.

[14] Smith MJ, Perrett DI, Jones BC (2006). Facial appearance is a cue to oestrogen levels in women. *Proceedings of the Royal Society B*.

[15] Shackelford TK, Larsen RJ (1999). Facial attractiveness and physical health. *Evolution and Human Behavior*.

[16] Roberts SC, Little AC, Gosling LM (2005). MHC-heterozygosity and human facial attractiveness. *Evolution and Human Behavior*.

[17] Thornhill R, Gangestad SW (2006). Facial sexual dimorphism, developmental stability, and susceptibility to disease in men and women. *Evolution and Human Behavior*.

[18] Coetzee V, Perrett DI, Stephen ID (2009). Facial adiposity: a cue to health?. *Perception*.

[19] Coetzee V, Chen J, Perrett DI, Stephen ID (2010). Deciphering faces: quantifiable visual cues to weight. *Perception*.

[20] Carré JM, McCormick CM, Mondloch CJ (2009). Facial structure is a reliable cue of aggressive behavior: research report. *Psychological Science*.

[21] Carré JM, McCormick CM (2008). In your face: facial metrics predict aggressive behaviour in the laboratory and in varsity and professional hockey players. *Proceedings of the Royal Society B*.

[22] Pound N, Penton-Voak IS, Surridge AK (2009). Testosterone responses to competition in men are related to facial masculinity. *Proceedings of the Royal Society B*.

[23] Strelau J (2002). *Temperament, A Psychological Perspective*.

[24] Patwardhan B, Warude D, Pushpangadan P, Bhatt N (2005). Ayurveda and traditional Chinese medicine: a comparative overview. *Evidence-Based Complementary and Alternative Medicine*.

[25] Song IB (2005). *An Introduction to Sasang Constitutional Medicine*.

[26] Lee J, Jung Y, Yoo J, Lee E, Koh B (2009). Perspective of the human body in sasang constitutional medicine. *Evidence-Based Complementary and Alternative Medicine*.

[27] Lee SW, Jang ES, Lee J, Kim JY (2009). Current researches on the methods of diagnosing sasang constitution: an overview. *Evidence-Based Complementary and Alternative Medicine*.

[28] Lee EJ, Sohn EH, Yoo JH (2005). The study of Sasangin’s face. *Journal of Sasang Constitutional Medicin*.

[29] Koo I, Kim JY, Kim MG, Kim KH (2009). Feature selection from a facial image for distinction of sasang constitution. *Evidence-Based Complementary and Alternative Medicine*.

[30] Seok JH, Song JH, Kim HJ (2007). An hardware error analysis of 3D automatic face recognition apparatus (3D-AFRA): surface reconstruction. *Journal of Sasang Constitutional Medicine*.

[31] Yoon JH, Lee SK, Lee EJ, Koh BH, Song IB (2000). Morphological standardization research of head and face on the 50’s and 60’s in Korean according to Sasang constitution. *Journal of Sasang Constitutional Medicine*.

[32] Jong MK, Shin YS, Kim YK (2006). A study on the bone mineral density (BMD) and body mass index (BMI) to the middle women based on the Sasang constitution. *Journal of Korean Clinical Nursing Research*.

[33] Chae H, Lyoo IK, Lee SJ (2003). An alternative way to individualized medicine: psychological and physical traits of Sasang typology. *Journal of Alternative and Complementary Medicine*.

[34] Shim EB, Lee SW, Kim SJ, Leem CH, Kwon YK, Baik YS (2009). Mitochondria hypothesis on the obesity-prone tendency in Tae-Eum people. *Korean Journal of Oriental Physiology & Pathology*.

[35] Ferrario VF, Sforza C (1997). Size and shape of soft-tissue facial profile: effects of age, gender, and skeletal class. *The Cleft Palate-Craniofacial Journal*.

[36] Park SH, Kim MG, Lee SJ, Kim JY, Chae H Temperament and character profiles of Sasang typology in an adult clinical sample.

[37] Steyerberg EW, Harrell FE, Borsboom GJJM, Eijkemans MJC, Vergouwe Y, Habbema JDF (2001). Internal validation of predictive models: efficiency of some procedures for logistic regression analysis. *Journal of Clinical Epidemiology*.

[38] R Development Core Team (2008). *A Language and Environment for Statistical Computing*.

[39] Fisher RA (1915). Frequency distribution of the values of the correlation coefficient in samples of an indefinitely large population. *Biometrika*.

[40] Wang D, Zhang W, Bakhai A (2004). Comparison of Bayesian model averaging and stepwise methods for model selection in logistic regression. *Statistics in Medicine*.

